# Absence of Bronzed Liver in Remittent Fever

**Published:** 1853-10

**Authors:** 


*Absence of Bronzed Liver in Remittent Fever.*—Dr. McGowen, of Memphis, Tenn., publishes in the Memphis Medical Recorder, for September, the "*post mortem* examination of a *case* that had died of the comatose form of the Remittent Fever," in which the *bronze color of the liver*, described by Dr. Stewardson and others, as characteristic of the disease, was not perceived. Prof. Wooten, of Memphis, adds to this case some remarks, in which he says: "Since Dr. Stewardson published his cases, we have examined about a half dozen, and in not a single one have we witnessed the *bronzed liver* as described by that gentleman."

